# NLRP12 Inflammasome Expression in the Rat Brain in Response to LPS during Morphine Tolerance

**DOI:** 10.3390/brainsci7020014

**Published:** 2017-02-06

**Authors:** Sulie L. Chang, Wenfei Huang, Xin Mao, Sabroni Sarkar

**Affiliations:** 1 Institute of NeuroImmune Pharmacology, Seton Hall University, 400 South Orange Avenue, South Orange, NJ 07079, USA; wenfei.huang@shu.edu (W.H.); ninnamao@yahoo.com.cn (X.M.); Sabroni.Sarkar@gmail.com (S.S.); 2 Department of Biological Sciences, Seton Hall University, 400 South Orange Avenue, South Orange, NJ 07079, USA

**Keywords:** morphine tolerance, NLRP12 inflammasome, LPS

## Abstract

Morphine, an effective but addictive analgesic, can profoundly affect the inflammatory response to pathogens, and long-term use can result in morphine tolerance. Inflammasomes are protein complexes involved in the inflammatory response. The nucleotide-binding oligomerization domain-like receptor (NLR) Family Pyrin Domain Containing (NLRP) 12 (NLRP12) inflammasome has been reported to have anti-inflammatory activity. In this study, we examined the expression of NLRP12 inflammasome related genes in the adult F344 rat brain in response to the bacterial endotoxin lipopolysaccharide (LPS) in the presence and absence of morphine tolerance. Morphine tolerance was elicited using the 2 + 4 morphine-pelleting protocol. On Day 1, the rats were pelleted subcutaneously with 2 pellets of morphine (75 mg/pellet) or a placebo; on Days 2 and 4 pellets were given. On Day 5, the animals were randomly assigned to receive either 250 µg/kg LPS or saline (i.p.). The expression of 84 inflammasome related genes in the rat brain was examined using a Ploymerase Chain Reaction (PCR) array. In response to LPS, there was a significant increase in the expression of the pro-inflammatory cytokine/chemokine genes interleukin-1 beta (Il-1β), interleukin-6 (Il-6), C-C motif chemokine ligand 2 (Ccl2), C-C motif chemokine ligand 7 (Ccl7), C-X-C motif chemokine ligand 1 (Cxcl1), and C-X-C motif chemokine ligand 3 (Cxcl3) and a significant decrease in the anti-inflammatory NLRP12 gene in both morphine-tolerant and placebo-control rats compared to saline-treated rats, although the changes were greater in the placebo-control animals. The Library of Integrated Network-Based Cellular Signatures’ (LINCS) connectivity map was used to analyze the list of affected genes to identify potential targets associated with the interactions of LPS and morphine tolerance. Our data indicate that, in the morphine tolerant state, the expression of NLRP12 and its related genes is altered in response to LPS and that the Vacuolar protein-sorting-associated protein 28 (VPS28), which is involved in the transport and sorting of proteins into sub-cellular vesicles, may be the key regulator of these alterations.

## 1. Introduction

Morphine is a potent analgesic that is widely used clinically for pain management. However, long-term use of morphine can lead to morphine tolerance and addiction [1]. In addition, morphine can profoundly and detrimentally affect the body’s immune system, at both the cellular and molecular levels. Morphine suppresses lymphocyte trafficking; decreases natural killer cell activity; inhibits the production of pro-inflammatory cytokines, such as tumor necrosis factor-alpha (TNF-α) and interleukin-1 beta (IL-1β) [2,3,4]; and induces atrophy of immune organs, such as the spleen and thymus [5,6,7,8,9]. 

Morphine-induced immunosuppression significantly increases the risk of bacterial infection [10]. Although the immunosuppressive effects of morphine have been widely studied, the mechanisms involved in the body’s inflammatory response to pathogens during morphine tolerance have not been fully investigated. 

Inflammasomes are multi-protein complexes assembled from nucleotide oligomerization domain receptor proteins known as nucleotide-binding oligomerization domain (NOD)-like receptors (NLR) [11]. They function as important mediators of innate immunity. Inflammasomes play a key role in the regulation of inflammation and immune responses by participating in the production of pro-inflammatory cytokines, including IL-1β and interleukin-18 (IL-18) [11,12,13]. Both IL-1β and IL-18 produce a wide variety of biological effects associated with infection, inflammation, and autoimmune processes.

There have been about 20 NLR inflammasomes identified in humans. The most commonly studied ones include NLR Family Pyrin Domain Containing 1 (NLPR1), NLR family apoptosis inhibitory protein 6 (NAIP2), NLR Family Pyrin Domain Containing 3 (NLRP3), NLR Family Pyrin Domain Containing 5 (NLRP5), NLR Family Pyrin Domain Containing 6 (NLRP6), NLR Family Pyrin Domain Containing 12 (NLRP12), NLR family member X1 (NLRPX1), and NLR Family CARD Domain Containing 4 (NLRC4) [14]. Inflammasomes have been sub-divided into two groups; pro-inflammatory inflammasomes and anti-inflammatory inflammasomes. The pro-inflammatory inflammasomes include NLRP3 and NLRC4, and their functions have been widely studied. The anti-inflammatory inflammasomes include NLRP12, NLRX1, NLRC3, and NLRC5. They appear to function by limiting or suppressing a pro-inflammatory response; however, this group has not been well studied to date. 

There have been a few recent reports characterizing the anti-inflammatory properties of NLRP12 [15,16]. NLRP12 can inhibit NF-κB signaling through both the canonical and non-canonical pathways, which are important in the control and regulation of innate immune responses [15,16]. NLRP12 inhibits IL-1 receptor-associated kinase 1 (IRAK1), a downstream component of the pathogen activated Toll-like receptor (TLR) pathway, which, in turn, decreases the signaling of the canonical NF-κB pathway [17]. In the non-canonical NF-κB pathway, NLRP12 interacts with and rapidly degrades NF-κB-inducing kinase (NIK), thereby suppressing NF-κB signaling [18]. 

The Library of Integrated Network-Based Cellular Signatures (LINCS) [19] is a database which implements a biological network-based strategy to make assessments regarding the impact of drugs, genetics, and related biological perturbations (alterations induced by external or internal mechanisms) on cellular states. The library database is based on the philosophy that typical human pathology, biology, and pharmacology are most aptly understood using a systems-level approach. It was constructed to generate a robust approach for perturbing a diversity of cell types, measuring cellular responses, integrating and analyzing data, and visualizing and interrogating the database for a variety of biomedical research applications [19]. This library allows researchers to access a wide variety of data by using a matrix consisting of cell type by experimental treatment by phenotypic assay. Using LINCS, researchers are able to inquire about information regarding mechanism-based relationships among the effects of different drug responses and their targets (perturbents) as well as associations among responding cellular components, in the format of network interactions and structure-function relationships [20].

In this study, we used an inflammasome PCR array containing 84 inflammasome-related genes to investigate the expression of inflammasomes during an inflammatory response to the bacterial endotoxin, lipopolysaccharide (LPS), in the rat brain during morphine tolerance. LINCS was then used to project possible candidate targets from the mRNA gene expression profiles generated from the PCR array analysis.

## 2. Materials and Methods

### 2.1. Animals

Fisher/NHsd 344 (F344) rats were purchased from Harlan Laboratories (Indianapolis, IN, USA). The animals were housed in groups of 3–5 animals in a temperature controlled (21 °C–22 °C) animal holding room, under a 12-h light/12-h dark illumination cycle (lights on at 7:00 a.m.). Food and tap water were provided ad libitum. The Institutional Animal Care and Use Committee (IACUC) at Seton Hall University, South Orange, NJ, USA approved the experimental protocol.

### 2.2. Morphine and LPS Administration

A 2 + 4 regimen was used to produce morphine tolerance in 7–8 mo old (250–350 g) male F344 rats [5,21,22,23,24]. The rats (*n* = 16) were randomly assigned into two groups. The morphine-tolerant group received two 75 mg morphine sulfate pellets (NIDA, Rockville, MD, USA) on Day 1 via subcutaneous (s.c.) implantation and four pellets on Day 2, whereas the control group received placebo pellets on both days. On Day 5, the two groups were randomly assigned to receive either LPS (250 µg/kg, Sigma, St. Louis, MO, USA) or saline (vehicle) [5,21,22,23,24]. Thus, the four experimental groups were placebo-control + saline, placebo-control + LPS, morphine-tolerant + saline, and morphine-tolerant + LPS. Two hours after the treatment with LPS or saline, the animals were euthanized and the brains were harvested.

### 2.3. RNA Isolation and Preparation of cDNA

Total RNA was extracted from the brain tissue using TRIZOL (Invitrogen, Carlsbad, CA, USA), following the manufacturer’s protocol. To remove contaminating DNA, the total RNA samples were treated with RNase-free DNase (Qiagen, Valencia, CA, USA), followed by further purification using an RNeasy Mini Kit (Qiagen, Valencia, CA, USA). The RNA quality and quantity were assessed using a nanodrop spectrophotometer (Thermo Scientific, Waltham, MA, USA). An equal amount of RNA (400 ng) from each sample was then converted into first-strand cDNA using a RT^2^ First Strand Kit (SABiosciences, Frederick, MD, USA) for a PCR array. 

### 2.4. Real-Time PCR Array

The expression of 84 key genes involved in the function of inflammasomes, general NLR signaling, and cytokine and chemokine genes was quantified using a custom PCR array and RT^2^ SYBR Green Fluorescein qPCR Master Mix (SABiosciences, Frederick, MD, USA), according to the manufacturer’s protocol. Using an ABI Prism 7900HT Fast Detection System (Applied Biosystems, Foster, CA, USA), real-time PCR was performed by first denaturing the PCR mix at 95 °C for 10 min, followed by 40 cycles at 95 °C for 15 s and at 60 °C for 1 min.

### 2.5. PCR Array Data Analysis

The expression of each gene was normalized to housekeeping genes and calculated using the ∆∆*C*t method. The threshold and baseline values were set manually, and the resulting threshold cycle values (*C*t) were analyzed using the PCR array data analysis template supplied on the manufacturer’s website [25]. The mean fold change in mRNA expression from 3 to 5 biological replicates was considered significant at *p* < 0.05. The gene profile signatures were created for every two groups compared.

### 2.6. LINCS Analysis

The differentially expressed genes were input into the Query App (apps.lincscloud.org/query), as described previously [26,27]. Based on the LINCS database, LincsCloud utilized gene profile signatures generated from the PCR array to generate a report, including probability outcomes in terms of gene knockdown effects and drug mimics. The scores given in the report evaluated how much a particular set of gene regulation features (named pertubagens) was likely to be connected with the genes listed in the LINCS report. Positive readings in the Consensus Knockdown Connections in the report indicate that knockdown of the genes listed in the LINCS report would match the gene changes input into the Query App, and thus the genes with high scores represent potential target genes for the experimental treatment.

## 3. Results

### 3.1. Expression Profile of Inflammasome-Related Genes Following an LPS Challenge, with and without Morphine Tolerance

**Figure 1:**
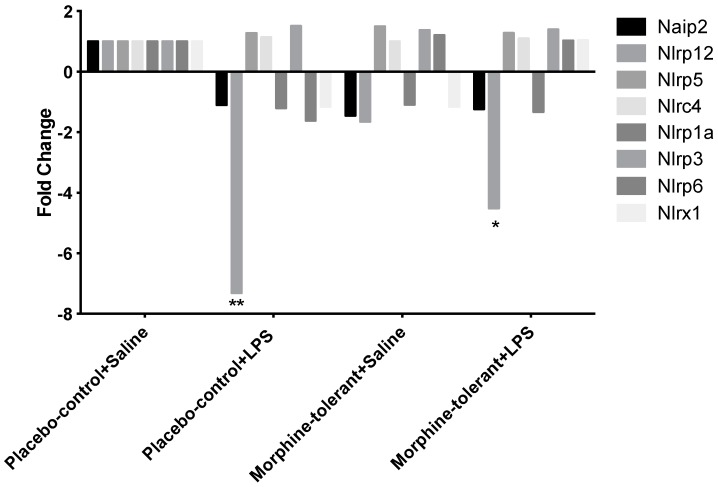
Inflammasome-related gene expression in the rat brain in response to lipopolysaccharide (LPS), with and without morphine tolerance. The expression of the inflammasome-related NOD-like receptor (NLR) genes (Naip2, Nlrp12, Nlrp5, Nlrc4, Nlrp1a, Nlrp3, Nlrp6, And Nlrx1) in the brains of rats given an i.p. injection of either 250 µg/kg LPS or saline, with and without morphine tolerance (*n* = 3–5 rats per group), was determined using a PCR array. Data were calculated using the ΔΔCT method, relative to the control group (placebo-control +saline), and are represented as a fold change. * *p* < 0.05, ** *p* < 0.01. Naip2: NLR family, apoptosis inhibitory protein 6; Nlrp12: NLR Family Pyrin Domain Containing 12; Nlrp5: NLR Family Pyrin Domain Containing 5; Nlrc4: NLR Family CARD Domain Containing 4; Nlrp1a: NLR family, pyrin domain containing 1A; Nlrp3: NLR Family Pyrin Domain Containing 3; Nlrp6: NLR Family Pyrin Domain Containing 6; Nlrpx1: NLR family member X1.

Alterations in gene expression were measured in rats challenged with LPS, with and without morphine tolerance, using a PCR array containing 84 genes related to inflammasome activation and function. NLRP12 expression was significantly decreased in response to LPS in both the morphine-tolerant (morphine-tolerant + LPS) and control (placebo-control + LPS) rats, compared to the rats given saline (morphine-tolerant + saline and placebo-control + saline) (Figure 1, Table 1). However, the decrease was greater in the placebo-control rats (−7.3 fold; *p* < 0.01) than in the morphine-tolerant rats (−4.5 fold; *p* < 0.05).

### 3.2. Expression Profile of Inflammasome-Related Downstream Signaling Genes Following an LPS Challenge, With and Without Morphine Tolerance

**Figure 2:**
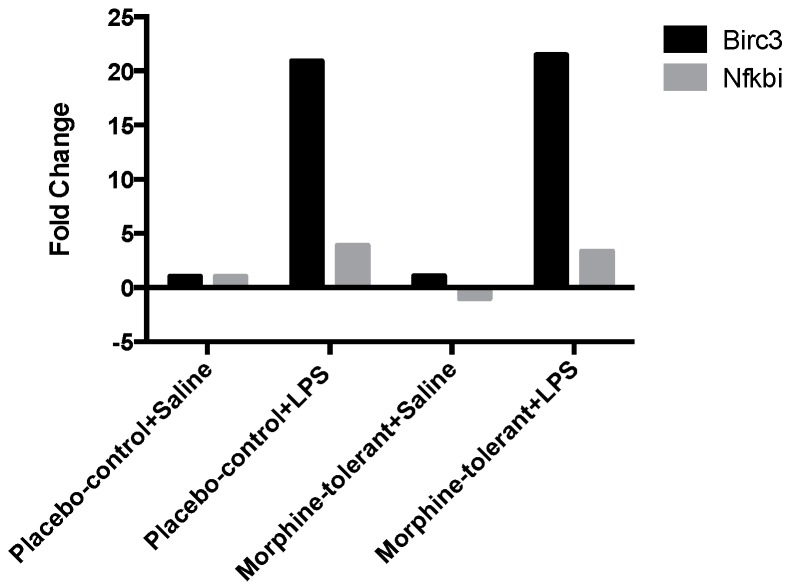
Inflammasome-related downstream gene expression in the rat brain in response to Lipopolysaccharides (LPS), with and without morphine tolerance. The expression of the inflammasome-related downstream signaling genes Baculoviral IAP Repeat-Containing 3 (Birc3) and NF-Kappa-B Inhibitor Alpha (Nfkbia) in the brains of rats given an i.p. injection of either 250 µg/kg LPS or saline, with and without morphine tolerance (*n* = 3–5 rats per group), was determined using a Polymerase Chain Reaction (PCR) array. The data were calculated using the ΔΔ*C*T method relative to the control group (placebo-control + saline) and are represented as fold change.

With a few exceptions, there were no significant changes in expression of the downstream signaling genes in response to LPS in either the morphine-tolerant rats (morphine-tolerant + LPS) or the control animals (placebo-control + LPS), compared to the rats given saline (morphine-tolerant + saline and placebo-control + saline) (Table 1). However, Baculoviral IAP Repeat-Containing 3 (Birc3), an important regulator gene involved in the downstream effects of inflammasomes, was significantly increased in response to LPS in both the morphine-tolerant (21.5 fold; *p* < 0.05) and control (21 fold; *p* < 0.01) groups, compared to the rats given saline (Figure 2, Table 2). In addition, NF-Kappa-B Inhibitor Alpha (Nfkbia), an inhibitor protein of NF-κB, was increased in both groups given LPS (morphine-tolerant + LPS, 3.4 fold, *p* < 0.01; placebo-control + LPS, 3.9 fold, *p* < 0.01) (Figure 2, Table 2).

### 3.3. Expression Profile of Inflammasome-Related Chemokine and Cytokine Genes after an LPS Challenge, with and without Morphine Tolerance

**Figure 3:**
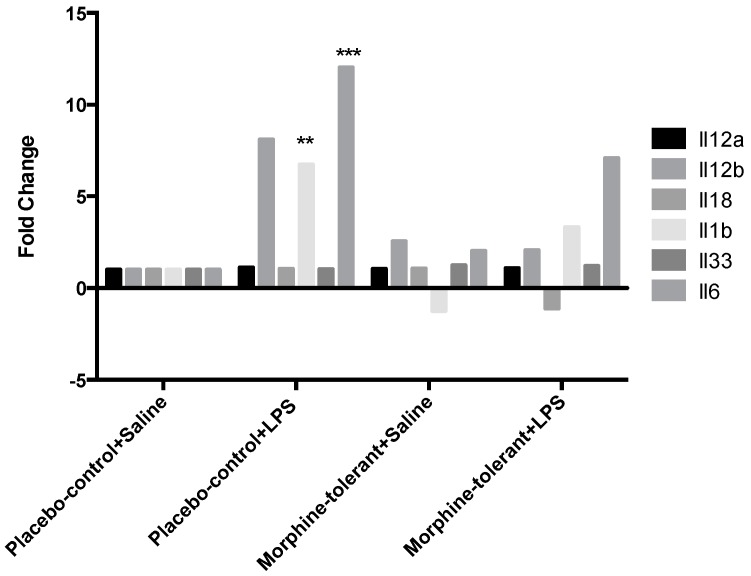
Cytokine gene expression in the rat brain in response to lipopolysaccharides (LPS), with and without morphine tolerance. Gene expression of interleukins Interleukin (Il)-1β, Il-6, Il-12a, Il-12b, Il-18, and Il-33 in the brains of rats, with and without morphine tolerance, following an i.p. injection of either 250 µg/kg LPS or saline (*n* = 3–5 rats per group) was determined using a Polymerase Chain Reaction (PCR) array. Data were calculated using the ΔΔCT method relative to the control group (placebo-control + saline) and are represented as a fold change. * *p* < 0.05, ** *p* < 0.01, *** *p* < 0.001

Cytokine and chemokine gene expression in response to LPS was greater in the control rats (placebo-control + LPS) compared to the morphine-tolerant animals (morphine-tolerant + LPS) (Table 3). The cytokines IL-1β and IL-6 were significantly increased 7- and 12-fold, respectively (*p* < 0.01–0.001), in the control rats given LPS (placebo-control + LPS), whereas in the morphine-tolerant group (morphine-tolerant + LPS) the fold changes were not statistically significant (3- and 7-fold, respectively) compared to the rats given saline (Figure 3, Table 3).

**Figure 4:**
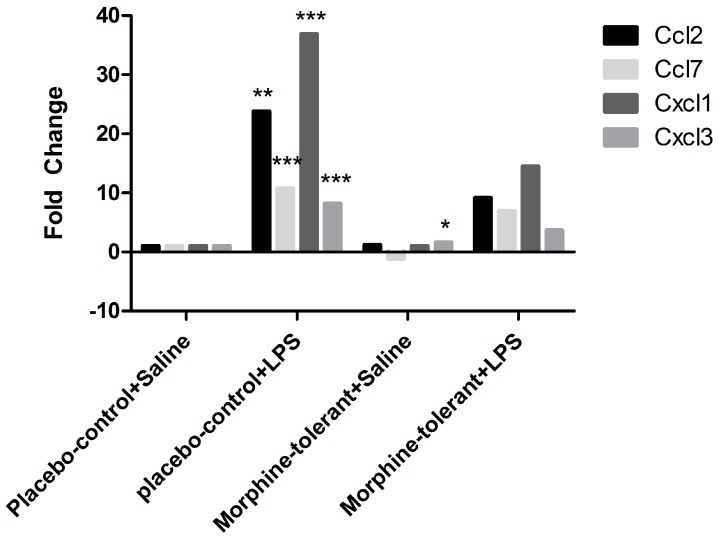
Chemokine gene expression in the rat brain in response to Lipopolysaccharides (LPS), with and without morphine tolerance. Gene expression of the chemokines C-C motif chemokine ligand (Ccl)2, Ccl5, Ccl7, Ccl11, Ccl12, C-X-C motif chemokine ligand (Cxcl)1, and Cxcl3 in the brains of rats with and without morphine tolerance, following an i.p. injection of either 250 µg/kg LPS or saline (*n* = 3–5 rats per group), was determined using a Polymerase Chain Reaction (PCR) array. Data were calculated using the ΔΔCT method relative to the control group (placebo-control + saline) and are represented as a fold change. * *p* < 0.05, ** *p* < 0.01, *** *p* < 0.001

Similarly, Ccl2, Ccl7, Cxcl1, and Cxcl3 chemokine expression was significantly increased 24-, 11-, 37-, and 8-fold, respectively (*p* < 0.01–0.001), in response to LPS in the control rats (placebo-control + LPS), whereas in the morphine-tolerant (morphine-tolerant + LPS) group the fold changes (9-, 7-, 14-, and 3-fold, respectively) were not statistically significant (Figure 4, Table 3).

### 3.4. LINCS Analysis of the Differentially Expressed Genes

Differentially expressed genes in the morphine-tolerant + saline versus morphine-tolerant + LPS rats and in the placebo-control + saline versus placebo-control + LPS rats as well as gene changes in rats the placebo-control + saline versus morphine-tolerant + saline rats were input into the Query App (apps.lincscloud.org/query). One report was generated by LINCS for each set of genes input. The genes with a high positive score in Consensus Knockdown Connections were considered to be potential gene targets (Table 4). In the placebo-control + saline versus placebo-control + LPS report, VPS28, protein C receptor (PROCR), and charged multivesicular body protein 2A (CHMP2A) were the top three with the highest scores. VPS28 is an ESCRT-I complex subunit that functions in the transport and sorting of proteins into sub-cellular vesicles. PROCR is endothelial protein C receptor involved in the blood coagulation pathway. CHMP2A is a component of the endosomal sorting complex required for transport III, which is involved in the degradation of surface receptor proteins and the formation of endocytic multivesicular bodies. 

In the placebo-control + saline versus morphine-tolerant + saline report, SWI/SNF related, matrix associated, actin dependent regulator of chromatin, subfamily e, member 1 (SMARCE1), aryl-hydrocarbon receptor repressor (AHRR), and glutathione peroxidase 7 (GPX7) were the most likely targets predicted by LINCS. SMARCE1 is required for the transcriptional activation of genes normally repressed by chromatin. AHRR mediates dioxin toxicity and is involved in the regulation of cell growth and differentiation. GPX7 is involved with cellular senescence and insulin secretion. 

In the morphine-tolerant + saline versus morphine-tolerant + LPS group, AHR (aryl hydrocarbon receptor), UBE2L6 (ubiquitin-conjugating enzyme E2L 6), and PAFAH1B3 (platelet-activating factor acetylhydrolase 1b, Catalytic Subunit 3) were the top three candidates. AHR is involved in the regulation of biological responses to planar aromatic hydrocarbons; UBE2L6 targets abnormal or short-lived proteins for degradation; and PAFAH1B3 functions in brain development and is associated with mental retardation, ataxia, and atrophy of the brain.

The predicted potential targets in each group were different from those in other groups, both in targets and their possibility rankings. VPS28 was the only one that appeared in both the Top 100 lists of placebo-control + saline versus placebo-control + LPS (No. 1 in Table 4) and morphine-tolerant + saline versus morphine-tolerant + LPS (No. 4 in Table 4).

Table 4 shows the top three potential target genes from each set of gene comparisons. There was no similarity in the gene rankings in the three sets of gene comparisons.

## 4. Discussion

Inflammasomes recognize a variety of pathogen-associated molecular patterns (PAMPs), including endotoxins such as LPS. Depending on the NLR proteins that constitute inflammasomes, an inflamasome can be pro-inflammatory or anti-inflammatory in nature [28]. For pro-inflammatory inflammasomes such as NLRP3, in vitro studies have shown that the activation and release of pro-inflammatory cytokines requires two signals. The first signal, triggered by PAMPs, leads to the activation of inflammasomes, which then provide the second signal. The activated inflammasomes, through caspase 1 activation, promote the production of the pro-inflammatory cytokines, IL-1β and IL-18. However, the signaling pathways during infection or inflammation in vivo are not yet completely defined [29], and the characteristics of anti-inflammatory inflammasomes such as NLRP12 have not yet been extensively investigated. To our knowledge, our study is one of the first to report the modulation of NLRP12 expression in response to LPS and morphine in vivo.

Recently, NLRP12 was designated as an anti-inflammatory NLR inflammasome protein. It is believed to be a negative regulator of the NF-κB signaling pathway by inhibiting downstream signaling of TLRs, particularly IRAK-1 [28,30]. Our results showed that NLRP12 expression decreased in the brains of both the control (placebo-control + LPS) and morphine-tolerant (morphine-tolerant + LPS) rats in response to an LPS challenge, indicating that one of the mechanisms by which LPS induces an inflammatory response is by inhibiting the expression of the anti-inflammatory NLRP12 inflammasome.

Although NLRP12 expression was decreased in both groups given LPS, the decrease was significantly greater in the control rats than in the morphine-tolerant rats, which suggests that the LPS-induced NLRP12 decrease is countered during morphine tolerance. Hence morphine may also modulate NLRP12 activity, directly or indirectly, thereby exerting its immunosuppressive effects and opposing the LPS-induced decrease in NLRP12 in the presence of morphine tolerance.

Birc3, a downstream regulator of inflammasome signaling, is essential for controlling the synthesis of cytokines and chemokines in the inflammatory Mapk and NF-κB pathways. It is also required for inflammasome activation, subsequent caspase 1 activity, and IL-1β formation [31]. In our study, Birc3 expression was significantly increased in both the placebo-control and morphine-tolerant rats in response to LPS, indicating that LPS is able to induce an inflammatory response through Birc3 activity, following inhibition of the anti-inflammatory NLRP12. However, in response to LPS, Birc3 expression in the morphine-tolerant rats did not change in comparison to the placebo-control rats. This indicates that morphine may not be able to modulate Birc3 expression, and therefore there is no change, increase or decrease, in its expression in the morphine tolerant state.

NF-κB is important in the activation of inflammatory mediators such as cytokines and chemokines [16]. Previous studies have reported that NLRP12 inhibits both canonical and non-canonical NF-κB activation [16,28] and that Nfkbia, a downstream regulator of inflammasomes, inhibits the activity of dimeric NF-κB/Rel complexes [32]. In our study, Nfkbia was significantly increased in response to LPS in both the placebo-control and morphine-tolerant rats. During an inflammatory response, one would expect the expression and activity of a positive regulator of inflammation to be increased, whereas that of a negative regulator would be decreased. However, from a physiological standpoint, there is a constant effort to balance pro- and anti-inflammatory activity [33,34]. This quest to balance the pro- and anti-inflammatory responses could be one of the reasons for an increase in Nfkbia, which is known to inhibit the activity of the pro-inflammatory dimeric NF-κB/REL, thus reducing the production of pro-inflammatory mediators.

As expected, we found that the expression of pro-inflammatory cytokines (IL-1β and IL-6) and chemokines (Ccl2, Ccl7, Cxcl1, and Cxcl3) was increased in response to LPS in the placebo-control rats [35,36,37]. In the morphine-tolerant rats, however, the LPS-induced cytokine and chemokine expression levels were lower, suggesting that NLRP12 inhibition in response to LPS may be opposed or subdued in the morphine tolerant state. 

In a previous study, we observed that, in peripheral immune organs such as the spleen, NLRP3 expression, but not NLRP12 expression, is altered in response to LPS, with and without morphine tolerance [38], suggesting that the mechanism(s) of inflammasome activation in response to pathogens may be different in peripheral immune organs, compared to the central nervous system. During morphine tolerance, the LPS-induced expression of NLRP3, as well as that of cytokines and chemokines, is reduced in comparison to the placebo-control rats given LPS [38]. These observations are consistent with previous studies showing that immune activation, including an inflammatory response, is diminished during morphine tolerance [39]. Therefore, the data from the present study, as well as from our previous report [38], collectively indicate that morphine may exert its effects through both pro- and anti-inflammatory inflammasomes.

LINCS analysis is able to predict potential target genes based on a certain treatment and the gene profile signatures in its database. In our study, LINCS was able to generate a report of potential targets with a *p* value of <0.05 from the list of genes with altered expression in response to LPS in control rats (placebo-control + saline versus placebo-control + LPS) but not from the other two comparisons (placebo-control + saline versus morphine-tolerant + saline, morphine-tolerant + saline versus morphine-tolerant + LPS), because there were not enough significant gene features in those two groups. When enlarging the set of gene features for the comparison of placebo-control + saline versus placebo-control + LPS to a *p*-value of <0.1, LINCS generated a report with similar potential targets. Thus, the gene features were then studied with a *p*-value of <0.1 on all three sets of comparisons. VPS28, PROCR, and CHMP2A were the top three with the highest scores in LINCS report generated based on placebo-control + saline versus placebo-control + LPS gene alternations, suggesting that Vps28, Procr and Chmp2a were potential targets of LPS. In the report for placebo-control + saline versus morphine-tolerant + saline, SMARCE1, AHRR, and GPX7 were the most likely targets altered in morphine tolerance predicted by LINCS. In the morphine-tolerant + saline versus morphine-tolerant + LPS report, AHR, UBE2L6, and PAFAH1B3 were the top three candidates that potentially responsible for the LPS-induced immune responses during morphine tolerance in rats. In the LINCS reports, the listed potential targets had different rankings using the different sets of gene features. This confirms that the response to LPS by those inflammosome-related genes could be affected by morphine tolerance. 

Moreover, among the targets listed above, while VPS28 was No. 1 in the control rats in response to LPS, it was No. 4 in morphine-tolerant rats (Table 4). The VPS28 protein functions in transporting and sorting proteins into sub-cellular vesicles. In our study, LINCS analysis suggests that the actions of VPS28 in response to LPS could be dampened during morphine tolerance.

## 5. Conclusions

The results from our study indicate that, in the rat brain, LPS-induced inflammation involves both the inhibition of the NLRP12 anti-inflammatory inflammasome and the stimulation of downstream regulators such as Birc3, thereby increasing the expression of pro-inflammatory chemokines and cytokines. However, in the morphine tolerant state, the response to LPS is dampened, as indicated by the reduced expression of inflammasome-related genes. LINCS analysis confirmed that the response to LPS is altered during morphine tolerance and indicated that VPS28 may be one of the genes responsible for the alterations associated with morphine tolerance.

## Figures and Tables

**Table 1:** Expression profile of inflammasomes and NLR genes in the rat brain in response to lipopolysaccharides (LPS), with and without morphine tolerance.

	morphine-tolerant + saline/placebo control + saline		placebo control + LPS/placebo control + saline		morphine-tolerant + LPS/morphine-tolerant + saline	
Gene	Fold Change	*p*-value *	Fold Change **	*p*-value *	Fold Change **	*p*-value *
Card6	−1.2347	0.078926	−1.5292	0.004159	−1.714	0.017499
Casp1	1.1505	0.299299	1.2132	0.276593	1.2764	0.02969
Casp12	1.0972	0.462347	1.5422	0.003614	1.4249	0.092085
Casp8	−1.0402	0.612597	−1.1363	0.391056	−1.2652	0.139999
Naip2	−1.4483	0.067329	−1.1031	0.619799	−1.2316	0.25758
Nlrp12	−1.6504	0.674422	−7.31	0.002731	−4.5124	0.025372
Nlrp5	1.494	0.054876	1.274	0.20786	1.2819	0.027352
Nlrc4	1.0028	0.915792	1.1349	0.159712	1.1003	0.445719
Nlrp1a	−1.0898	0.470844	−1.2027	0.001147	−1.3308	0.1379
Nlrp3	1.3773	0.134647	1.5114	0.054565	1.3956	0.02407
Nlrp6	1.205	0.420019	−1.6225	0.358997	1.0293	0.784547
Nlrx1	−1.1574	0.24832	−1.1494	0.191026	1.039	0.679959
Nod2	−1.6257	0.650152	1.0901	0.811946	1.2826	0.50395
Pycard	−1.0575	0.610143	−1.0285	0.797363	−1.2363	0.030115

* For *p*-value, letters in red mean *p* < 0.05. ** For Fold Change, letters in red mean fold change >2 and letters in blue mean the fold change <−2. For the color lines, 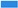: 6–10 fold decrease; 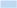: 2–5 fold decrease; 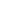: <2 fold; 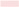: 2–5 fold increase; 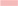: 6–10 fold increase; 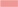: 11–30 fold increase; 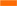: 31–50 fold increase.

**Table 2:** Expression profile of inflammasome-related downstream signaling genes in the rat brain in response to lipopolysaccharides (LPS), with and without morphine tolerance. Full name of the genes were provided in Appendix Table A1.

	morphine-tolerant + saline/placebo control + saline		placebo control + LPS/placebo control + saline		morphine-tolerant + LPS/morphine-tolerant + saline	
Gene	Fold Change	*p*-value *	Fold Change **	*p*-value *	Fold Change **	*p*-value *
Bcl2	1.0469	0.863233	1.0586	0.845254	1.2398	0.339246
Bcl2l1	−1.1256	0.01111	1.0572	0.476643	−1.0291	0.671184
Birc2	1.0509	0.586337	−1.0281	0.622487	1.0699	0.531417
Birc3	1.0648	0.587513	20.9356	0.011065	21.5025	0.001873
Cflar	1.0226	0.697346	1.3021	0.004748	1.2155	0.140978
Chuk	1.1867	0.119108	−1.0233	0.754891	1.1593	0.073058
Ciita	−1.0617	0.750879	1.0619	0.873363	1.4678	0.39646
Ctsb	−1.0009	0.978499	−1.0656	0.617885	−1.1046	0.493204
Fadd	−1.1261	0.258884	1.1235	0.252486	1.0306	0.71174
Hsp90aa1	1.2774	0.114349	1.0391	0.743375	1.189	0.149024
Hsp90ab1	1.0752	0.376876	−1.0968	0.305427	1.1579	0.18515
Ikbkb	1.0718	0.550623	−1.0011	0.962992	1.1799	0.201955
Ikbkg	1.0924	0.324904	−1.0402	0.492725	1.1419	0.079202
Irak1	1.1549	0.062648	1.0287	0.755395	1.2213	0.242955
Map3k7	−1.2495	0.002404	−1.1008	0.434596	−1.2497	0.114056
Map3k7ip1	−1.0335	0.664499	−1.1151	0.369226	1.02	0.864431
Map3k7ip2	−1.2869	0.000867	−1.1169	0.456355	−1.3427	0.007785
Mapk1	−1.0527	0.468581	−1.0887	0.159097	1.0316	0.724595
Mapk11	−1.0673	0.530192	−1.0706	0.578016	−1.0633	0.571223
Mapk12	1.1424	0.420684	1.1889	0.372287	−1.0255	0.780819
Mapk13	−1.1629	0.924422	−1.5423	0.355826	1.0504	0.651078
Mapk14	1.0018	0.940725	−1.0318	0.81795	1.0335	0.709962
Mapk3	−1.1294	0.224192	−1.121	0.372958	−1.0495	0.76638
Mapk8	1.0383	0.883566	−1.0215	0.798267	1.0037	0.911563
Mapk9	−1.1252	0.03886	−1.0075	0.87718	−1.0805	0.288977
Mefv	1.2572	0.278807	1.2582	0.463496	1.9192	0.028283
Myd88	1.0211	0.791137	1.0103	0.917416	1.2185	0.03802
Nfkb1	1.1098	0.279116	1.2919	0.089881	1.3323	0.033357
Nfkbia	−1.0294	0.835021	3.885	0.001215	3.3744	0.001348
Nfkbib	−1.1473	0.330475	−1.0153	0.762037	−1.1167	0.643504
P2rx7	−1.1586	0.428492	1.0742	0.625549	1.0578	0.661519
Panx1	−1.076	0.275575	1.1455	0.406863	1.1101	0.334734
Pea15a	−1.0943	0.291531	−1.043	0.625202	−1.0802	0.492135
Pstpip1	−1.1041	0.527175	−1.1018	0.680263	−1.0232	0.87342
Ptgs2	−1.08	0.499364	1.5126	0.000571	1.1762	0.323717
Rage	1.0729	0.478448	−1.1273	0.17423	1.1854	0.16574
Rela	1.0171	0.804744	1.1801	0.417806	1.2504	0.106174
Ripk2	−1.1057	0.237762	1.2913	0.028006	1.1199	0.389288
Sugt1	1.0063	0.918273	−1.1023	0.519396	−1.1264	0.106262
Tirap	−1.3098	0.094434	−1.0971	0.535721	1.0809	0.374146
Hsp90b1	1.0475	0.680071	−1.0334	0.69801	−1.0877	0.314466
Traf6	−1.0344	0.915204	−1.0171	0.943304	1.0988	0.437323
Txnip	−1.0761	0.70951	−1.0718	0.699702	−1.0187	0.789181
Xiap	1.0572	0.503462	−1.0558	0.471524	1.0089	0.93573

* For *p*-value, letters in red mean *p* < 0.05. ** For Fold Change, letters in red mean fold change >2 and letters in blue mean the fold change <−2. For the color line, 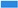: 6–10 fold decrease; 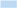: 2–5 fold decrease; 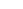: <2 fold; 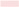: 2–5 fold increase; 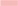: 6–10 fold increase; 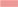: 11–30 fold increase; 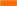: 31–50 fold increase.

**Table 3:** Expression profile of the cytokine and chemokine genes in the rat brain in response to LPS, with and without morphine tolerance.

	morphine-tolerant + saline/placebo control + saline		placebo control + LPS/placebo control + saline		morphine-tolerant + LPS/morphine-tolerant + saline	
Gene	Fold Change **	*p*-value *	Fold Change **	*p*-value *	Fold Change **	*p*-value *
Ccl11	−2.4662	0.023104	−2.8639	0.16576	−2.7346	0.249229
Ccl12	−1.4362	0.646529	2.0214	0.219015	2.1108	0.399119
Ccl2	1.1756	0.521412	23.7845	0.001354	9.1497	0.106064
Ccl5	−1.105	0.472434	1.2909	0.290174	−1.0949	0.766893
Ccl7	−1.0841	0.688167	10.7188	0.000259	6.9528	0.163034
Cxcl1	1.0131	0.944854	36.8609	0	14.4718	0.130008
Cxcl3	1.6208	0.019673	8.1909	0.000076	3.6477	0.170486
Cd40lg	−2.2796	0.215753	1.1119	0.704126	−2.4054	0.273798
Ifnb1	1.2519	0.936419	1.6899	0.391302	1.5695	0.513801
Ifng	1.5355	0.290413	1.1698	0.767882	2.2615	0.088346
Il12a	1.05	0.57557	1.1178	0.33855	1.0837	0.456191
Il12b	2.5587	0.26828	8.1055	0.102341	2.077	0.198782
Il18	1.068	0.60015	1.0484	0.707002	−1.1151	0.48359
Il1b	−1.2476	0.284501	6.7366	0.001383	3.3136	0.089312
Il33	1.2342	0.054615	1.0407	0.583823	1.1978	0.072613
Il6	2.023	0.45937	12.0303	0.008058	7.0943	0.083969
Irf1	1.1299	0.521156	3.4575	0.000386	3.4104	0.01276
Irf2	−1.1481	0.050782	1.0027	0.916481	1.0556	0.593809
Irf3	−1.2824	0.024657	−1.3225	0.225481	−1.2478	0.088625
Irf4	−1.1491	0.43182	−1.0557	0.846866	1.0263	0.858979
Irf5	−1.1024	0.38296	−1.1468	0.376953	1.0391	0.841777
Irf6	1.1055	0.413463	1.012	0.792025	1.118	0.272222
Tnfsf11	−1.6077	0.800779	−1.362	0.519436	−1.3266	0.965379
Tnfsf14	−1.396	0.243156	−1.8445	0.126355	−1.4278	0.629476
Tnfsf4	−1.0175	0.997398	−1.0887	0.790199	−1.3422	0.420711

* For *p*-value, letters in red mean *p* < 0.05. ** For Fold Change, letters in red mean fold change >2 and letters in blue mean the fold change <−2. For the color lines, 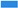: 6–10 fold decrease; 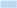: 2–5 fold decrease; 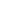: <2 fold; 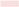: 2–5 fold increase; 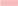: 6–10 fold increase; 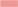: 11–30 fold increase; 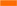: 31–50 fold increase.

**Table 4:** LINCS Consensus Knockdown Connections from differentially expressed genes in the rat brain in response to lipopolysaccharides (LPS), with and without morphine tolerance. (A) Top 10 Consensus Knockdown Connections in the three sets of gene comparisons; (B) Rankings of the top three Consensus Knockdown Connections in the three sets of gene comparisons. Full name of the genes were provided in Appendix Table A2.

(A)	**Rank**	**placebo-Saline vs. Placebo-LPS**	**Placebo-Saline vs. Morphine-Tolerant-Saline**	**Morphine-Tolerant-Saline vs. Morphine-Tolerant-LPS**
	1	VPS28	SMARCE1	AHR
	2	PROCR	AHRR	UBE2L6
	3	CHMP2A	GPX7	PAFAH1B3
	4	MB	ATP5F1	VPS28
	5	ZNF768	CALR	JUNB
	6	RBPJ	GPR110	RYK
	7	WARS2	CHMP2A	ARG1
	8	TBX2	ELF4	PROC
	9	MRPS2	FGFR1	ZNF324
	10	MAP3K14	F7	ATP5D
(B)	**Gene Rank**	**Placebo-Saline vs. Placebo-LPS**	**Placebo-Saline vs. Morphine-Tolerant-Saline**	**Morphine-Tolerant-Saline vs. Morphine-Tolerant-LPS**
	VPS28	1	42	4
	PROCR	2	491	681
	CHMP2A	3	7	177
	SMARCE1-1	675	1	504
	AHRR	2383	2	460
	GPX7	488	3	2137
	AHR	46	769	1
	UBE2L6	89	545	2
	PAFAH1B3	95	622	3

## Appendix A

**Table A1:** Genes analyzed in PCR Array.

Gene Symbol	Full Name	mRNA Entry
Card6	caspase recruitment domain family, member 6	NM_001106413.1
Casp1	caspase 1	NM_012762.2
Casp12	caspase 12	NM_130422.1
Casp8	caspase 8	NM_022277.1
Naip2	NLR family, apoptosis inhibitory protein 6	XM_008760697.2
Nlrp12	NLR family, pyrin domain containing 12	NM_001169142.1
Nlrp5	NLR family, pyrin domain containing 5	NM_001107474.1
Nlrc4	NLR family, CARD domain containing 4	NM_001309432.1
Nlrp1a	NLR family, pyrin domain containing 1A	NM_001145755.2
Nlrp3	NLR family, pyrin domain containing 3	NM_001191642.1
Nlrp6	NLR family, pyrin domain containing 6	NM_134375.3
Nlrx1	NLR family member X1	NM_001025010.1
Nod2	nucleotide-binding oligomerization domain containing 2	NM_001106172.1
Pycard	PYD and CARD domain containing	NM_172322.1
Bcl2	BCL2, apoptosis regulator	NM_016993.1
Bcl2l1	BCL2 like 1	NM_001033670.1
Birc2	baculoviral IAP repeat-containing 2	NM_021752.2
Birc3	baculoviral IAP repeat-containing 3	NM_023987.3
Cflar	CASP8 and FADD-like apoptosis regulator	NM_001033864.2
Chuk	conserved helix-loop-helix ubiquitous kinase	NM_001107588.1
Ciita	class II, major histocompatibility complex, transactivator	NM_001270803.1
Ctsb	cathepsin B	NM_022597.2
Fadd	Fas associated via death domain	NM_152937.2
Hsp90aa1	heat shock protein 90, alpha (cytosolic), class A member 1	NM_175761.2
Hsp90ab1	heat shock protein 90 alpha family class B member 1	NM_001004082.3
Ikbkb	inhibitor of kappa light polypeptide gene enhancer in B-cells, kinase beta	NM_053355.2
Ikbkg	inhibitor of kappa light polypeptide gene enhancer in B-cells, kinase gamma	NM_199103.1
Irak1	interleukin-1 receptor-associated kinase 1	NM_001127555.1
Map3k7	mitogen activated protein kinase kinase kinase 7	NM_001107920.2
Map3k7ip1	TGF-beta activated kinase 1/MAP3K7 binding protein 1	NM_001109976.2
Map3k7ip2	TGF-beta activated kinase 1/MAP3K7 binding protein 2	NM_001012062.1
Mapk1	mitogen activated protein kinase 1	NM_053842.2
Mapk11	mitogen-activated protein kinase 11	NM_001109532.2
Mapk12	mitogen-activated protein kinase 12	NM_021746.1
Mapk13	mitogen activated protein kinase 13	NM_019231.2
Mapk14	mitogen activated protein kinase 14	NM_031020.2
Mapk3	mitogen activated protein kinase 3	NM_017347.2
Mapk8	mitogen-activated protein kinase 8	NM_053829.2
Mapk9	mitogen-activated protein kinase 9	NM_001270544.1
Mefv	Mediterranean fever	NM_031634.1
Myd88	myeloid differentiation primary response 88	NM_198130.1
Nfkb1	nuclear factor kappa B subunit 1	NM_001276711.1
Nfkbia	NFKB inhibitor alpha	NM_001105720.2
Nfkbib	NFKB inhibitor beta	NM_030867.2
P2rx7	purinergic receptor P2X 7	NM_019256.1
Panx1	Pannexin 1	NM_001270548.1
Pea15a	phosphoprotein enriched in astrocytes 15	NM_001013231.1
Pstpip1	proline-serine-threonine phosphatase-interacting protein 1	NM_001106824.2
Ptgs2	prostaglandin-endoperoxide synthase 2	NM_017232.3
Rage	MOK protein kinase	NM_001010965.1
Rela	RELA proto-oncogene, NF-kB subunit	NM_199267.2
Ripk2	receptor-interacting serine-threonine kinase 2	NM_001191865.1
Sugt1	SGT1 homolog, MIS12 kinetochore complex assembly cochaperone	NM_001013051.1
Tirap	TIR domain containing adaptor protein	XM_017596001.1
Hsp90b1	heat shock protein 90 beta family member 1	NM_001012197.2
Traf6	TNF receptor associated factor 6	NM_001107754.2
Txnip	thioredoxin interacting protein	NM_001008767.1
Xiap	E3 ubiquitin-protein ligase XIAP	NM_022231.2
Ccl11	C-C motif chemokine ligand 11	NM_019205.1
Ccl12	chemokine (C-C motif) ligand 12	NM_001105822.1
Ccl2	C-C motif chemokine ligand 2	NM_031530.1
Ccl5	C-C motif chemokine ligand 5	NM_031116.3
Ccl7	C-C motif chemokine ligand 7	NM_001007612.1
Cxcl1	C-X-C motif chemokine ligand 1	NM_030845.1
Cxcl3	C-X-C motif chemokine ligand 3	NM_138522.1
Cd40lg	CD40 ligand	NM_053353.1
Ifnb1	interferon beta 1	NM_019127.1
Ifng	interferon gamma	NM_138880.2
Il12a	interleukin 12A	NM_053390.1
Il12b	interleukin 12B	NM_022611.1
Il18	interleukin 18	NM_019165.1
Il1b	interleukin 1 beta	NM_031512.2
Il33	interleukin 33	NM_001014166.1
Il6	interleukin 6	NM_012589.2
Irf1	interferon regulatory factor 1	NM_012591.1
Irf2	interferon regulatory factor 2	NM_001047086.1
Irf3	interferon regulatory factor 3	NM_001006969.1
Irf4	interferon regulatory factor 4	NM_001106108.1
Irf5	interferon regulatory factor 5	NM_001106586.1
Irf6	interferon regulatory factor 6	NM_001108859.1
Tnfsf11	tumor necrosis factor superfamily member 11	NM_057149.1
Tnfsf14	tumor necrosis factor superfamily member 14	NM_001191803.1
Tnfsf4	tumor necrosis factor superfamily member 4	NM_053552.1

**Table A2:** Top 10 scored Genes in LINCS report.

Gene Symbol	Full name
VPS28	VPS28, ESCRT-I subunit
PROCR	protein C receptor
CHMP2A	charged multivesicular body protein 2A
MB	myoglobin
ZNF768	zinc finger protein 768
RBPJ	recombination signal binding protein for immunoglobulin kappa J region
WARS2	tryptophanyl tRNA synthetase 2 (mitochondrial)
TBX2	T-box 2
MRPS2	mitochondrial ribosomal protein S2
MAP3K14	mitogen-activated protein kinase kinase kinase 14
SMARCE1	SWI/SNF related, matrix associated, actin dependent regulator of chromatin, subfamily e, member 1
AHRR	aryl-hydrocarbon receptor repressor
GPX7	glutathione peroxidase 7
ATP5F1	ATP synthase, H+ transporting, mitochondrial Fo complex subunit B1
CALR	calreticulin
GPR110	G protein-coupled receptor 110
ELF4	E74 like ETS transcription factor 4
FGFR1	fibroblast growth factor receptor 1
F7	coagulation factor VII
AHR	aryl hydrocarbon receptor
UBE2L6	ubiquitin-conjugating enzyme E2L 6
PAFAH1B3	platelet-activating factor acetylhydrolase 1b, catalytic subunit 3
JUNB	JunB proto-oncogene, AP-1 transcription factor subunit
RYK	receptor-like tyrosine kinase
ARG1	arginase 1
ZNF324	zinc finger protein 324
ATP5D	ATP synthase, H+ transporting, mitochondrial F1 complex, delta subunit
